# Understanding the Effect of Graphene Nanoplatelet Size on the Mechanical and Thermal Properties of Fluoroelastomer-Based Composites

**DOI:** 10.3390/polym17182534

**Published:** 2025-09-19

**Authors:** Santiago Maldonado-Magnere, Mehrdad Yazdani-Pedram, Pablo Fuentealba, Andrónico Neira-Carrillo, Miguel A. Lopez-Manchado, Hector Hernandez-Villar, Allan Bascuñan-Heredia, Mohamed Dahrouch, Héctor Aguilar-Bolados

**Affiliations:** 1 Facultad de Química y de Farmacia, Pontifica Universidad Católica de Chile, Casilla 306, Santiago 6094411, Chile; 2 Facultad de Ciencias Químicas y Farmacéuticas, Universidad de Chile, Olivos 1007, Santiago 8380544, Chilepfuentealbacastro@ciq.uchile.cl (P.F.); 3 Facultad de Ciencias Veterinarias y Pecuarias, Universidad de Chile, Santa Rosa 11735, Santiago 8820808, Chile; aneira@uchile.cl; 4 Instituto de Ciencia y Tecnología de Polímeros, ICTP-CSIC, Juan de la Cierva, 328006-Madrid, Spain; 5 Departamento de Polímeros, Facultad de Ciencias Químicas, Universidad de Concepción, Concepción 3349001, Chileabascunan2017@udec.cl (A.B.-H.); 6 Departamento de Química Orgánica, Facultad de Ciencias Químicas, Universidad de Concepción, Concepción 3349001, Chile; mdahrouch@udec.cl

**Keywords:** fluoroelastomer, graphite and graphene nanoplatelets, mechanical properties, thermal properties

## Abstract

This study presents a comprehensive evaluation of the behavior of fluoroelastomer (FKM) compounds reinforced with graphene nanoplatelets of various sizes such as 15 μm (GN15) and 5 μm (GN5). The study evaluates the mechanical, dynamic mechanical, thermal, wetting, and photothermal properties of the compounds when irradiated with an 808 nm laser. The results demonstrate that the size of the graphene nanoplatelets significantly impacts the mechanical properties, with smaller sizes exhibiting a stronger reinforcing effect compared to larger nanoplatelets. Additionally, clear evidence of an influence on dynamic mechanical properties was observed, particularly through the broadening of the damping factor (tan δ) peak. This suggests modifications to the material’s viscoelastic behavior. Regarding the photothermal response, it was found that smaller nanoplatelets (GN5) dispersed in the rubber matrix allow higher temperatures to be reached and thermal equilibrium to be achieved more efficiently under irradiation. Overall, the results suggest that FKM compounds containing graphene nanoplatelets can attain high temperatures with low-energy infrared irradiation. This makes them promising materials for technological applications in extreme environments, such as the Arctic, high mountains, or space, where materials with controlled thermal responses and high mechanical performance are required.

## 1. Introduction

Recent advances in polymer nanocomposites highlight the importance of optimizing filler size, dispersion, and processing strategies to tailor multifunctional properties to demanding industrial applications. Graphene-based composites have been shown to significantly improve mechanical strength, thermal stability, and surface properties in both elastomeric and thermoplastic matrices when properly designed through process optimization and hybrid strategies [1,2,3,4]. For example, Subramani et al. demonstrated the role of graphene reinforcement in optimizing the mechanical performance of 3D-printed impellers by adjusting the process parameters of composites based on polylactic acid (PLA) and graphene [5], while hybrid approaches based on machine learning and simulation have also enabled the sustainable manufacture of high-performance FDM (Fused deposition modeling) composites [6]. Beyond structural reinforcement, graphene-based materials have also been exploited for tribological and multifunctional applications, including surface engineering and energy absorption [6]. Similarly, recent work on bio-based nanocomposites designed using 3D printing shows that interactions between the filler and the matrix critically determine the performance, sustainability, and application-specific reliability [7,8]. This knowledge reinforces the need to systematically investigate the influence of filler morphology and particle size, such as graphene nanoplates, on the viscoelastic, thermal, and photothermal response of elastomeric systems such as fluoroelastomers, where this knowledge is still limited. Indeed, graphene materials have also attracted interest due to their high cross-section absorption in the NIR region [9,10,11]. Applications for this property are diverse, including photothermal therapy for cancer treatment and solar energy storage [12,13].

The above raises the question: is it possible to obtain elastomeric materials that exhibit a thermal response to low-energy irradiation? To answer this question, it must be taken into account that graphene materials are suitable for this purpose and that the fluoroelastomers are among the elastomers with the highest thermal stability. Indeed, FKM elastomers have unique and reliable properties; for example, they have high chemical resistance, excellent mechanical properties, and a wide operating temperature range. This is because FKM contains carbon-fluorine bonds in its structure [14,15]. In addition, fluoroelastomers have curing systems (diamine or bisphenol) that increase resistance at room temperature and improve their service temperature range without sacrificing flexibility, which differentiates them from other types of elastomers [16]. Another question that arises is whether the morphological characteristics of the graphene material used affect its thermal response to irradiation. This is a relevant concern due to the wide variety of graphene-based materials available on the market or that can be obtained through experimentation [17].

Based on the above, this work is focused on the study of FKM-based compounds containing graphene nanoplatelets of different sizes with the aim of evaluating the influence of the characteristics of graphene nanoplatelets on a wide variety of properties, such as mechanical, mechano-dynamic, and surface properties. This topic remains poorly explored. This work aims not only to provide a more complete understanding of graphene-based and FKM-based systems, but also to develop high-performance elastomeric materials of interest to the automotive, aerospace, energy, and oil industries [14,18,19].

## 2. Materials and Methods

### 2.1. Materials

The fluoroelastomer used was FKM C90, supplied by Jiangsu FreChem Co., Ltd. (Wuxi, China). As filler, 100 mesh graphite powder (>80%), and graphene nanoplatelets (5 μm and 15 μm particle size) were used, which were purchased from Sigma-Aldrich (St. Louis, MO, USA) and used without further purification. Magnesium(II) oxide, calcium(II) hydroxide, 2,2-bis(4-hydroxyphenyl) hexafluoropropane (Bisphenol AF), and benzyl triphenylphosphonium chloride (BPP) were also purchased from Sigma-Aldrich (St. Louis, MO, USA).

### 2.2. Preparation of Composites

500 g of FKMC90 was processed for 5 min to achieve homogenization using two rollers separated by 1 mm. Then, predetermined amounts of magnesium(II) oxide and calcium(II) hydroxide were gradually added, followed by the filler. The mixture was then processed for a further 20 min to ensure uniform dispersion. The curing agents, bisphenol AF and BPP, were then incorporated and the mixture was processed for another 10 min. Table 1 shows the formulations of the prepared composites. The filler content, consisting of 10 phr of graphite (G) and graphene nanoplatelets (GN), was chosen based on the carbon black content typically used in conventional rubber composites [20].

**Figure 1:**
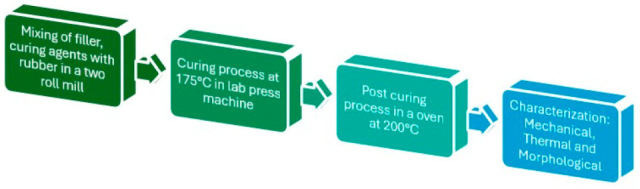
Infographic illustrating the process of rubber nanocomposite manufacturing.

The cross-linking process of the composites was carried out in laboratory hydraulics with heating plates model ZL-3022, Zhongli Instrument Technology Co., Ltd. (Dongguan, China). The conditions of cross-linking time, mixing speed and temperature conditions were determined by a moving matrix rheometer model ZL-3001, Zhongli Instrument Technology Co. Ltd. (Dongguan, China) (Figure 1). The selected conditions were 175 °C for 10 min to prepare the cross-linked composites. These composites were then subjected to post-crosslinking at 200 °C for 24 h in a Memmert oven (Buchenbach, Germany).

### 2.3. Characterization of FKM Composites

The FKM composites and their respective fillers were characterized by X-ray diffraction (XRD) analysis with a Bruker model D8 Advance diffractometer (Bruker Co., Billerica, MA, USA) equipped with a Cu Kα radiation source (*λ* = 0.154 nm), power supply 40 kV and 40 mA. The angle of incidence (2*θ*) varied between 2° and 80°, and the scanning speed was 0.02 degrees/s. Interlayer distances were determined by studying the reflection of the (002) plane in the graphite sample (G) and in the graphene nanoplatelets of 5 μm (GN5) and 15 μm (GN15) using Bragg’s law (Equation (1)) [21].

(1) $$d_{\left(00l\right)}=\frac{\lambda }{2sin\theta_{\left(00l\right)}}$$

where *θ*_(00l)_ is the reflection angle of the reflection plane and 001 is an integer number and *d*_(00l)_ is the interlayer distance.

The particle size ($L_{\left(00l\right)}$) the different graphitic materials was determined by the Debye−Scherrer equation (Equation (2)) [22].

(2) $$L_{\left(00l\right)}=\frac{\kappa \lambda }{{\beta cos\theta }_{\left(00l\right)}}$$

where κ is the Scherrer constant (κ = 0.89), *λ* is the X-ray wavelength (1.5417 Å), *β* corresponds to the full width at half-maximum (FWHM) of the peak, and *θ*_00l_ is the reflection angle.

The number of stacked layers were calculated using the Equation (3).

(3) $$N_{L}=\frac{d_{\left(00l\right)}}{L_{\left(00l\right)}}+1$$

The fillers and composites were also characterized by Raman spectroscopy using a Renishaw (Renishaw plc, Wotton-under-Edge, UK) Invia Raman microscope equipped with a 514.5 nm wavelength laser and 0.02 cm^−1^ resolution. The spectra were recorded from 0 to 4000 cm^−1^, and each Raman spectrum was normalized with respect to its highest intensity band. The interdefect distance was determined using the Equation (4), which was proposed by Cançado et al. [23].

(4) $$\frac{I_{D}}{I_{G}}=C_{A}\cdot \frac{\lambda_{L}^{4}}{L_{D}^{2}}$$

where *I_D_*/*I_G_* corresponds to intensity ratio of D and G bands registered, *C_A_*, 1350 cm^−1^ and 1580 cm^−1^, respectively. *C_A_* constant depends on the laser excitation energy (4.2 × 10^−10^ nm^−4^ for a laser with wavelength of 514 nm), and *λ* is the laser wavelength.

The cure curves of the compounds were determined using a moving die rheometer (model ZL-3001, Zhongli Instrument Technology Co., Ltd., Dongguan, China) at 175 °C. The analysis was performed according to GB/T 16584 and ISO 6504 standards [24,25]. ~4 g of material was weighed and placed between two polyester sheets in a rheometer chamber for 15 min at 175 °C; the oscillation arc was 1.0° and the cavity die frequency was 1.7 Hz. All specimens were measured in triplicate. Mechanical properties were determined using an Instron (Instron Co., Nothwood, MA, USA) Model 3366 dynamometer at 25 °C with a crosshead speed of 500 mm/min, equipped with a 0.5 kN load cell (ASTM D412) [26]. Samples were measured in quintuplicate and the coefficient of variation of the values obtained was less than 10%. Compression set (ASTM D395) and Shore A hardness (ASTM D2240) were also determined [27,28].

The morphology of the FKM and FKM composites samples was analyzed using a Philips (Amsterdam, The Netherlands) XL30 ESEM scanning electron microscope (SEM). The samples were coated with an ultrathin gold layer. The SEM was operated at an accelerating voltage of 10 kV.

Thermal properties were determined by thermogravimetric analysis (TGA) measurements using a Netzsch (Selb, Germany) model Iris TG 209 F1 thermogravimetric analyzer under a nitrogen atmosphere at a heating rate of 10 °C/min. The operating temperature range was set at 25 to 900 °C. Differential scanning calorimetry (DSC) of the different FKM composites was performed using a Netzsch DSC 214 (Selb, Germany) in the range between −100 °C and 25 °C with a heating rate of 10 °C/min.

Dynamic mechanical analysis (DMA) of FKM and FKM composites was performed using a TA Instruments^®^ (New Castle, DE, USA) Model Q800 Dynamic Mechanical Analyzer in tensile mode at 1 Hz, over a temperature range of −50 °C to 50 °C, with a heating rate of 2 °C/min and an experimental error of less than 5%.

The contact angles of the FKM and FKM-based composite samples were obtained using a Dataphysics OCA 15EC optical contact angle system (DataPhysics Instruments GmBH, Filderstadt, Germany). The dosing volume was 16 µL. Average values are reported with standard deviation (± SD) after six replicates for each sample. For the study of thermal response, a Fluke TiS20 handheld thermographic camera ^®^ (Fluke Co., Everett, WA, USA) was used, with a temperature measurement accuracy of ±2 °C or 2%. The laser used was the Laserglow^®^ (Laserglow Technologies, Toronto, Canada) LRD-0808 series, with a wavelength of 808 nm. Measurements were conducted under six different conditions, varying the distance between the laser and the sample at 2 and 5 cm with minimum, medium, and maximum laser power levels corresponding to 1, 5, and 10, respectively. Measurement times were set at 1, 5, and 10 min with the laser applied. The distance between the camera and the sample was kept at 10 cm throughout.

Solid-state UV spectra were recorded using PerkinElmer (PerkinElmer, Inc., Waltham, MA, USA) Lambda 650 equipment coupled with an integration sphere consisting of a Praying Mantis™ diffuse reflection accessory and a “sampling kit” (model DRP-SAP), manufactured by Harrick Scientific Products, Inc. (New York, NY, USA). The band gap value was estimated by extrapolating the first linear region of the Tauc plots *(αhv)*^2^ vs. *hν*, where α is the absorption coefficient.

## 3. Results and Discussion

### 3.1. Graphene Nanoplatelets Characterization

The G, GN5 and GN15 materials were characterized using two essential techniques for understanding their structural characteristics: X-ray diffraction (XRD) and Raman spectroscopy.

**Figure 2:**
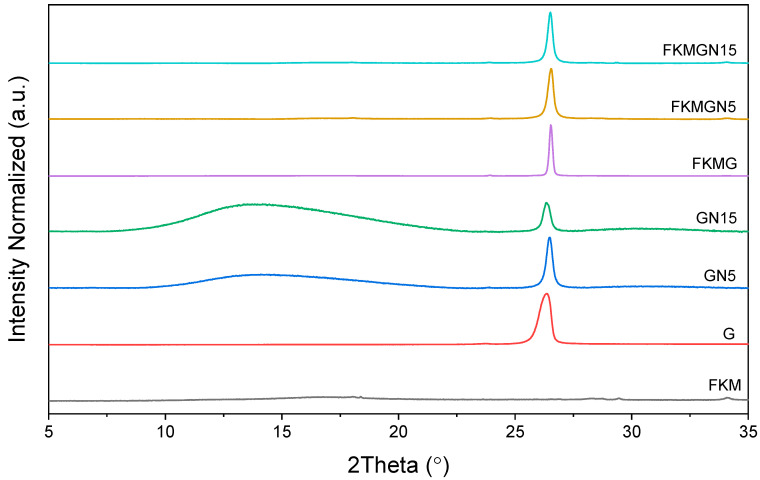
X-ray diffractogram of the nanoparticles and FKM-based composites.

Figure 2 shows the X-ray diffractograms of graphite (G) and graphene nanoplatelets with particle sizes below 5 μm (GN5) and 15 μm (GN15), materials used as fillers in FKM-based composites. As seen in Figure 2, the most relevant diffraction peak for these graphitic materials is that related to the reflection plane (002), which is associated with the interlayer distance between the graphitic layers [21,29].

For graphite (G), this peak is at 26.3° (*d*_002_ = 0.334 nm), while for GN5 and GN15, it appears at 26.3° (*d*_002_ = 0.334 nm) and 26.4° (*d*_002_ = 0.333 nm), respectively. The particle sizes (*L*) of the graphitic fillers were determined using Equation (2). It was found that the crystallite sizes for G, GN5 and GN15 are 16.0, 28.5 and 22.4 nm, respectively. By considering the equation of number of stacked layers, *N_L_* (Equation (3)), it is possible to estimate that G, GN5 and GN15 are composed of 48, 86 and 68 layers, respectively (Table 2).

In addition, it can be observed that in the diffractograms of GN5 and GN15 there are intense and broad signals, lower than 20°, which would correspond to exfoliated graphene nanoplatelets. Therefore, it is possible to conclude that both GN5 and GN15 have a contribution of graphite platelets as well as graphene, with GN15 being the one that contributes a greater proportion of graphene platelets. No amorphous phase contribution can be observed for graphite.

**Figure 3:**
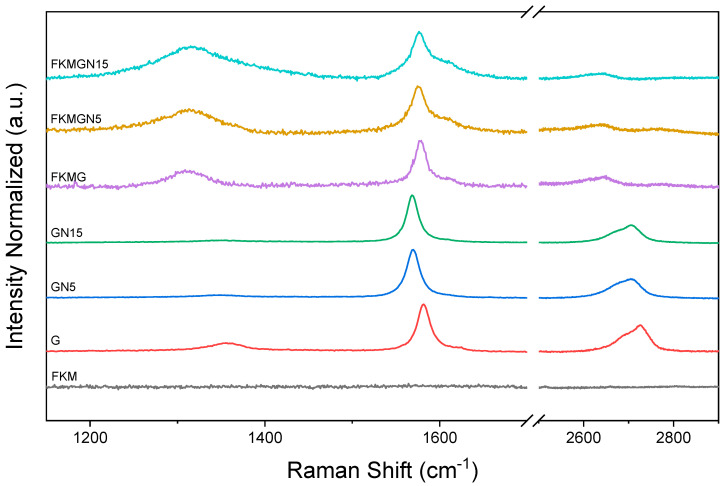
Raman shift of FKM and FKM composites.

Figure 3 shows the Raman spectra of nanoparticles and their FKM composites. For the graphite fillers, a low intensity absorption band is observed at about 1356 cm^−1^, known as the D band, which corresponds to defects in the graphitic structure attributed to functional groups at the edges. In addition, a band at 1582 cm^−1^, known as the G band, is observed, corresponding to the first-order vibrational mode of the E_2g_ phonon from the sp^2^ hybridization of carbon atoms in the graphitic sheet. A broad band is observed at 2692 cm^−1^, corresponding to the second order vibrational mode of the D band, known as the 2D band, and the overtone D + G is observed at 2728 cm^−1^ [21].

Table 3 shows the Raman shifts and intensities of the bands identified for each sample. In addition, the *I_D_*/*I_G_* parameter, known as the defect density, is presented and the *L_D_* parameter, corresponding to the crystallite size, is included. The *I*_2*D*_/*I_G_* parameter useful for estimating the number of stacked layers of graphene materials is also included. As observed, G has a higher *I_D_*/*I_G_* value than those of GN5 and GN15, which explains its low *L_D_*. This would be explained by the fact that the shorter distance between edge defects observed in G, attributed to its high layer stacking, implies high defect density greater than that observed for GN5 and GN15. The *L_D_* values of GN5 and GN15 do not show significant differences between them. It is important to note that the *I*_2*D*_/*I_G_* has low values for G, GN5, and GN15. This indicates that the graphitic materials used to prepare the nanocomposites present high values of stacked layers [23,30,31]. However, the mentioned parameters, *I_D_*/*I_G_* and *L_D_*, change significantly when these materials are incorporated as fillers in the FKM matrix. As seen in Table 3, the defect density increases significantly, while the crystal size decreases for all the graphitic materials studied. This can be explained by the mixing process in two roll mill, which would have affected the material, causing degradation and thus reducing the spacing between the defects. On the other hand, the observation of significant shifts of the D band, between 30 and 40 cm^−1^ with respect to the materials used as fillers, can be attributed to the interaction between the fluorine functions of FKM, which would polarize the six-membered rings responsible for the breathing mode vibrations that activate the D band. The latter can be seen in the 2D band, which is unfolded, and the D + G band, which is shifted up to 50 cm^−1^. It has been reported that the D-band of fluorinated graphenes shows a shift to lower frequencies, which has been attributed to defects other than graphene edge defects. It has been speculated that since graphene is constituted by networks of six-membered sp^2^-bonded carbon atom rings, the D band is mainly activated by Fluorine atoms acting as vacancies in these sp^2^ rings [15]. All the above allows us to conclude that the mixing process affects the quality of the graphenic materials, increasing its defects and decreasing the distance between them, also promoting a higher affinity of the polymeric matrix with the graphitic materials due to the interaction of the polarization of the pi systems with the fluorine groups of the FKM [16].

### 3.2. Mechanical Properties of FKM Composites

**Figure 4:**
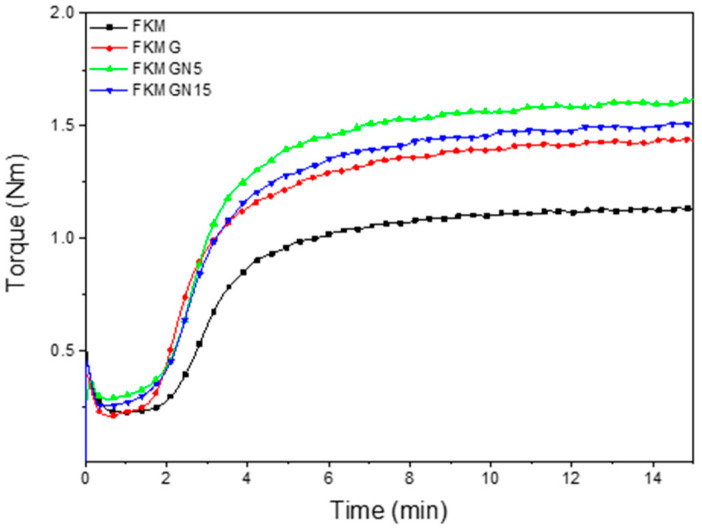
Curing curves of FKM and FKM-based composites.

Figure 4 presents the curing curves of FKM composites. Table 4 displays the values of parameters, such as minimum torque (*M_L_*), maximum torque (*M_H_*), scorch time (*t_s_*_2_) and optimum curing time (*t*_90_). It is observed for M_L_ that the use of graphite did not present significant variations in its initial torque compared to the unfilled FKM, with no effect of the amount of filler used on the viscosity being appreciated. However, the use of graphene nanoplatelets (GN) showed an improvement in the rigidity of the material, compared to the unfilled fluoropolymer, of 28.1% and 12.9% for GN5 and GN15, respectively. This is attributed to a greater capacity for stiffness transfer to the composite system, given by the lower number of graphitic sheets that these materials present compared to graphite, which allows their greater dispersion and matrix-filler interaction [1,32]. Regarding the M_H_ of the composites with 10 phr of filler, it was observed that the maximum torques presented a significant increase in their values compared to the FKM without filler (Table 4). The use of graphite (G), GN5 and GN15 showed an increase of +25.0%, +53.9% and +30.2% of *M_H_* values, respectively, compared to that of unfilled FKM. The high torque value achieved with GN5 is due to the fact that it has a smaller grain size than the other materials used as fillers. It has previously been demonstrated that graphene nanoplatelets in fluorosilicones and FKM elastomer have an effect on the curing curve, shifting it to higher torque values due to their reinforcing effect. Effects associated with a reduction in vulcanization time have been linked to the influence of the morphology and heat transport of graphene nanoplatelets, which affects the cross-linking process [3,16].

**Figure 5:**
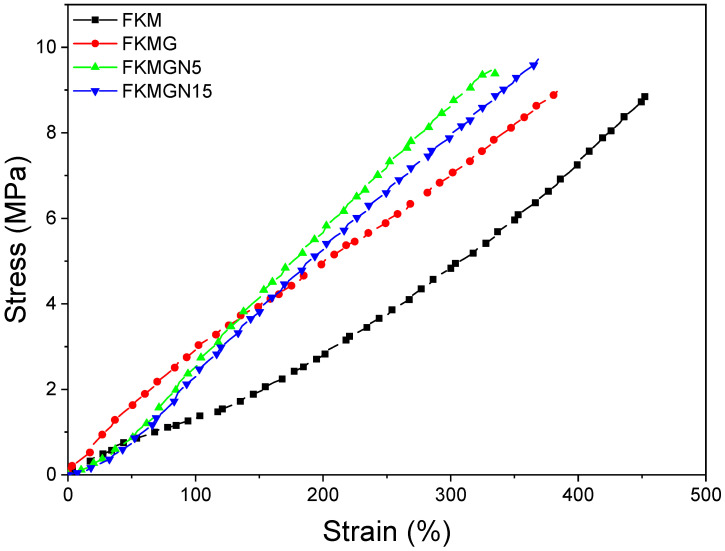
Tensile properties of FKM and FKM composites.

Figure 5 shows the stress–strain curves for FKM, FKMG, FKMGN5, and FKMGN15 composites. The FKM displayed a characteristic curve of materials with elastomeric behavior. This is also evident in the experimental results for elongation at break, which for this fluoropolymer was 477% deformation. For FKMGN5, the highest increase in tensile strength was observed at 330%, representing an approximately 31% decrease compared to unfilled FKM. GN 5 can transfer its rigidity to the cross-linked FKM system due to its smaller particle size and fewer stacked layers. For the FKMGN15 and FKMG composites, decreases in deformation of around 24% and 20% were recorded, respectively. This behavior is to be expected given the high rigidity of the filler, which in combination with its laminar morphology, restricts the displacement of chains at the molecular level and acts as a defect point that facilitates breakage [33,34,35].

Table 5 shows the values of the elastic modulus for FKM and FKM-based composites. For FKM, E50, E100, and E300 moduli of 0.69 MPa, 1.21 MPa, and 4.40 MPa were observed, respectively. These low modulus values correspond to elastomeric materials. For composites with 10 phr of filler, a marked increase in elastic modulus is observed. The highest E50 and E100 values, compared to unfilled polymer, were for FKMG, increasing by +113% and +135%, respectively. For E300, FKMGN5 showed the highest modulus increase of +101%. The increase in modulus indicates the ability of the graphitic sheets to impart greater rigidity to the composites while maintaining the elastomeric characteristics of the materials. For comparison, Saha et al. reported an increase in Young’s modulus of 140% and 220% for a loading of 4 phr of organically modified montmorillonite (Cloisite 30B) and graphene oxide, respectively [36]. In fact, the shape of graphene significantly increases the stiffness of various materials [37,38], a phenomenon also observed in important elastomers such as FKM.

Values of compression set and hardness of FKM and its composites are presented in Table 5. The hardness of FKMG and FKMGN5 composites increased moderately, by 7.94% and 11.6%, respectively, compared to FKM. The highest increase was observed for FKMGN15, with 25.2%. These values indicate a semi-reinforcement of the FKM matrix, attributed to the formation of an interlaminar layer with mechanical properties like the laminar fillers [3].

Moreover, the compression set results show that the FKM-G composite exhibited the highest value, with an increase of 311% compared to FKM. Similarly, the compression set values for FKMGN5 and FKMGN15 increased by 278% and 286%, respectively. These increases can be attributed to the laminar shape of the graphitic fillers, which induce permanent deflection in the materials. Moreover, high compression set values suggest stronger affinity between the matrix and the fillers, leading to reduced elasticity of the fluoroelastomer. This effect on compression set performance is attributed to the lamellar shape of the filler, which would facilitate an irreversible shape change in the graphite-reinforced FKM-based composites [16].

### 3.3. Dynamic Mechanical Analysis and Thermal Properties

**Figure 6:**
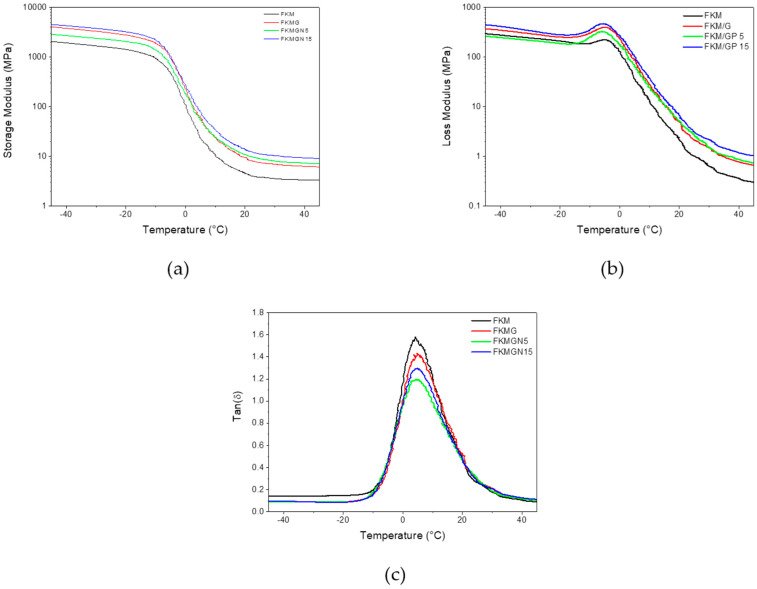
(**a**) Storage modulus, (**b**) loss modulus, and (**c**) damping factors of FKM and FKM composites.

Figure 6 presents the storage moduli, loss moduli and damping factors of the FKM and FKM-based composites containing G, GN5 and GN15. It is observed that the storage modulus of FKM experiences a temperature decrease ranging from −14 °C to 24 °C. This behavior is attributed to the transition from the glassy state to the elastomeric state, with the maximum loss occurring ca. 5.0 °C (Figure 6b). The glass transition temperatures (T_g_) of the FKM and FKM-based composites were obtained from the maximum damping factor (Tan delta) and their values were 4.2 °C, 5.1 °C, 4.8 °C and 4.9 °C for FKM, FKMG, FKMGN5 and FKMGN15, respectively. The shift to higher temperature of T_g_ can be attributed to the confinement effect of graphene nanolayers on polymer chains.

Regarding the storage moduli of the FKMG, FKMGN5 and FKMGN15 composites, shown in Figure 6a,b, they are slightly higher than that of FKM, suggesting the reinforcement effect of graphitic filler. Table 6 presents the parameters of damping factor such as FWHM and tanδ_max_. FKM exhibits a high tanδ_max_ value, which indicates its greater ability to dissipate mechanical energy as heat compared to FKM-based composites. Lower tanδ_max_ values are observed for FKM composites, indicating lower molecular mobility due to restrictions imposed by the filler material. The change in FWHM (Full Width at Half Maximum) compared to FKM reveals that the addition of filler imparts heterogeneity. This is likely due to the formation of separate microphases, which alter the occurrence of cooperative, homogeneous transitions. The impact is progressive and related to particle size; GN05 is the filler material that most affects the transition process. This is attributed to the stiffness transfer capability to the fluoroelastomer chains due to the stacking of the graphitic layers [39,40].

**Figure 7:**
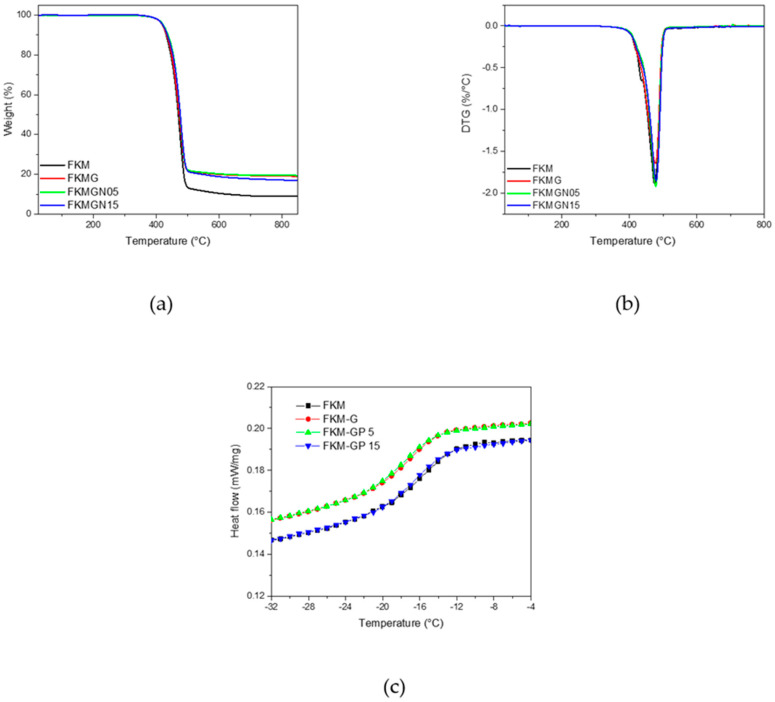
(**a**) thermogravimetric analysis (TGA), (**b**) first derivative of thermogravimetric analysis (DTG) and (**c**) differential scanning calorimetry (DSC) curves of FKM and FKM composites.

The thermogravimetric analysis (TGA) shown in Figure 7a demonstrated that FKM and FKM composites exhibited thermal stability up to approximately 330 °C. Beyond this temperature, a significant mass loss occurs in all composite materials and the fluoroelastomer. The maximum degradation temperature rate for all materials was around 475 °C, as indicated in the DTG (Figure 7b). This high temperature is attributed to the stability provided by the high fluorine content in the saturated polymer chains, which is approximately 66% for FKM. The degradation process, associated with the temperature of maximum mass loss, is due to the breaking of carbon-carbon bonds in the cross-linked polymer system. Table 4 summarizes the residual mass (%) of each sample, showing that composites with graphite and graphene nanoplatelet fillers leave an average mass residue of 18%.

On the other hand, Figure 7c shows the Differential Scanning Calorimetry analysis, from which the glass transition temperature can be obtained, which does not necessarily agree with that determined by the dynamic mechanical analysis (Table 7). The decrease in thermal flow, observed for FKMGN5 and FKMGN15 may be related to the fact that the incorporated filler has a higher thermal conductivity than the polymer. In fact, it has been reported that materials used as filler with different thermal conductivity dispersed in FKM elastomers affect the thermal conductivity and the heat flow observed in the DSC test for the resulting composites [16].

### 3.4. Morphological Analysis of FKM Composites

**Figure 8:**
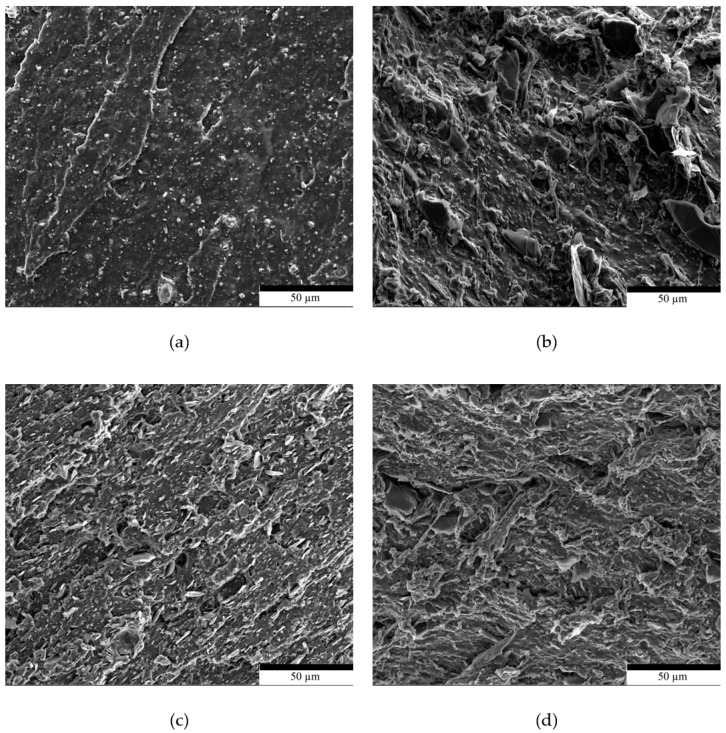
(**a**) Scanning electron microscope images of FKM, (**b**) FKMG, (**c**) FKMGN5, and (**d**) FKMGN15.

The scanning electron microscopy (SEM) images in Figure 8 show the cross sections of FKM tensile test specimens and their compounds after breakage. As can be seen, the FKM/G compounds with 10 phr of filler exhibit significant differences compared to unreinforced FKM. Figure 8b shows a noticeably rougher surface, which is attributed to poor dispersion of the filler in the polymer matrix during mixing.

By contrast, the micrographs of the FKMGN5 (Figure 8c) and FKMGN15 (Figure 8d) compounds reveal a more uniform and fine dispersion with less particle agglomeration than the samples using graphite as a filler. This suggests improved interfacial adhesion between the polymer matrix and the reinforcement. It is hypothesized that reducing the number of stacked graphene layers in the filler increases its compatibility with the matrix, thereby improving the mechanical properties.

### 3.5. Contact Angle and Thermal Response to Laser Infrared Arradiation

**Figure 9:**
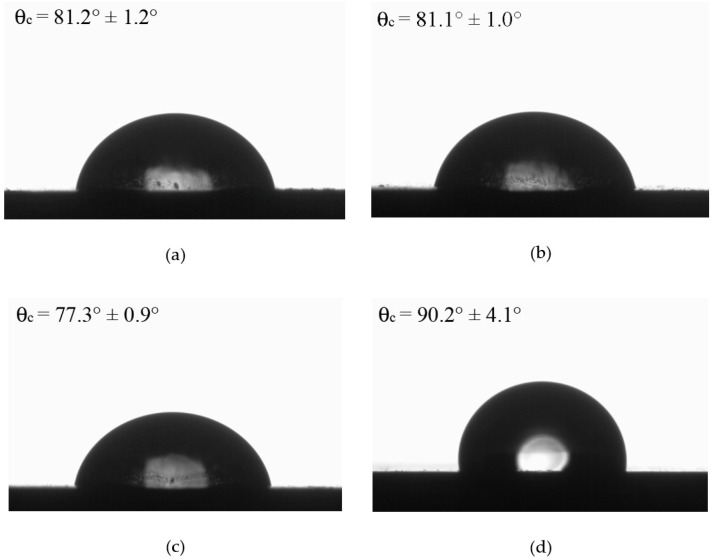
(**a**) Contact angle of FKM, (**b**) FKMG, (**c**) FKMGN5 and (**d**) FKMGN15.

Figure 9 shows the recorded images of the contact angle determination for FKM and the FKM-based composites. The average values, from at least six measurements per sample, were 81.2 ± 1.2, 81.1 ± 1.0, 77.7 ± 0.9 and 90.2 ± 4.1 for FKM, FKMG, FKMGN5 and FKMGN15, respectively. As can be seen, the effect of the fillers on the angle is low, especially those of FKMG and FKM05, but for FKMGN15 a slightly higher contact angle value is recorded. This difference can be attributed to the differences in roughness imparted by the characteristics of GN15, as well as to the polarity of this system. In addition, the high deviation of FKMGN15 contact angle value indicates that this material has higher surface heterogeneity [41].

Table 8, on the other hand, shows the temperatures recorded on the FKM surface and the FKM-based composites when irradiated with an 808 nm laser at different laser powers, distances and exposure times. The results clearly demonstrate that the incorporation of nanofillers, specifically graphitic materials, significantly enhances the photothermal response of the FKM elastomer composites. The heating is driven by the nanofillers’ ability to absorb infrared laser radiation and convert it into thermal energy. The dependence on laser power, time and distance can be identified. As expected, higher temperatures are reached as the laser power increases. Similarly, as the exposure time increases, the system approaches a steady state of equilibrium between the generated and lost heat. Conversely, increasing the distance results in a lower temperature being reached.

At short distance and high power, the FKM composites containing G and GN5 maximize the heating performance However, FKMGN15 shows a lower temperature increase at distance of 2 cm than the other samples. These results suggest that GN15 graphene nanoplatelets affect heat dissipation, which is related to the baseline shift identified in the DSC. This indicates that FKMGN15 can exhibit lower heating under aggressive conditions. In addition, FKMGN5 achieved the highest temperature, indicating that filler characteristics favor the formation of an efficient percolating network for photon absorption and heat transformation.

**Figure 10:**
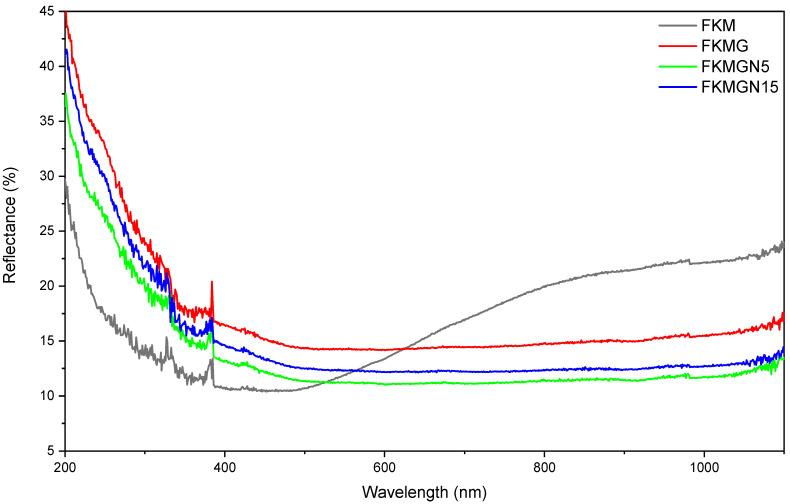
Reflectance spectra of FKM, FKMG, FKMGN5, FKM15.

The behavior of compounds containing graphite materials is due to their absorption properties in the visible and infrared. Figure 10 shows the reflectance spectra of the samples analyzed and the reflectance decreases due to the presence of the fillers. Specifically, at 808 nm, the %R decreases from 26.8 for FKM to 16.4 for FKMG, 14.1 for FKMGN15 and 12.8 for FKMGN5. The decrease in reflectance implies an increase in the absorption of electromagnetic radiation, which would be responsible for the greater increase in temperature of the samples. In turn, the decrease in reflectance observed in the FKM-based compounds could be due to different characteristics of the filler used, such as particle size, dispersion of the particles in the polymer matrix and photophysical properties.

The present results confirm that the size of graphene nanoplatelets play a decisive role in tailoring the viscoelastic and thermal performance of elastomer composites. This is primarily due to differences in interfacial adhesion and network formation within the FKM matrix. Similar size- and morphology-dependent reinforcement effects have been demonstrated in thermoplastic systems as well. For example, Raja et al. optimized graphene-enhanced PETG composites and found that improvements in storage modulus and thermal stability were strongly correlated with nanosheet dispersion and filler-matrix interactions [35]. Similarly, Subramani et al. reported that incorporating carbon-based reinforcements into PEEK significantly improved its structural performance when processing parameters were optimized systematically [42]. Beyond mechanical and thermal reinforcement, decision-making frameworks for carbon-filled composites have been introduced to ensure material reliability and application-specific selection [43]. Furthermore, broader reviews on polymer additive manufacturing emphasize that optimizing filler content, particle geometry, and processing strategies is crucial for balancing the mechanical performance, durability, and sustainability of advanced composites [44]. These findings reinforce the idea that the trends observed in FKM–GN composites are consistent with those in other high-performance polymer systems, in which filler morphology and dispersion dictate multifunctional behavior.

## 4. Conclusions

The particle size of graphene nanoplatelets strongly influences the performance of FKM compounds. Smaller nanoplatelets enhanced mechanical properties, including tensile strength and storage modulus, although elongation at break decreased due to restricted polymer chain mobility. Thermal stability improved, as the laminar morphology of the fillers slowed volatile diffusion and polymer degradation. The compounds also showed a remarkable photothermal response under 808 nm laser irradiation, with GN5-containing samples reaching the highest temperatures due to enhanced UV-Vis-NIR absorption.

These results demonstrate that FKM composites with graphene nanoplatelets are high-performance materials suitable for extreme environments, such as the Arctic, high mountains, or space. Future work could focus on optimizing nanoplatelet size and loading and evaluating performance under broader environmental conditions.

## Figures and Tables

**Table 1:** Composition of the prepared FKM composites.

Sample	FKM	FKMG	FKMGN5	FKMGN15
	phr *			
FKM	100	100	100	100
Magnesium(II) oxide	3	3	3	3
Calcium(II) hydroxide	6	6	6	6
Bisphenol AF	2	2	2	2
BPP	0.55	0.55	0.55	0.55
Graphite	0	10	0	0
Graphene nanoplatelets 5 nm	0	0	10	0
Graphene nanoplatelets 15 nm	0	0	0	10

* Part per hundred of rubber.

**Table 2:** Crystalline parameters of the nanoparticles and their FKM composites determined by Bragg’s and Debye Scherrer equation.

Parameter	G	GN5	GN15	FKMG	FKMGN5	FKMG15
2*θ* (°)	26.3	26.3	26.4	26.6	26.5	26.6
*d*_002_ (nm)	0.334	0.334	0.333	0.329	0.331	0.329
*L* (nm)	16.0	28.5	22.4	46.0	26.2	28.8
*N_L_*	48	86	68	140	80	87

**Table 3:** Raman shift of defect density (*I_D_*/*I_G_*), crystallite size (*L_D_*), Raman shift and intensity of G, GN5, GN15, FKMG, FKMGN5 and FKMGN15.

Band	Graphite		GN 5 um		GN 15 um		FKMG		FKMGN5		FKMGN15	
*I_D_*/*I_G_*	0.165		0.0471		0.0432		0.354		0.521		0.758	
*I*_2*D*_/*I_G_*	0.278		0.253		0.184		0.104		0.071		0.087	
							0.127		0.092		0.083	
*L_D_* (µm)	13.3		25.1		26.3		85.1		58.2		40.4	
	Raman shift (cm^−1^)	Intensity (a.u.)	Raman shift (cm^−1^)	Intensity (a.u.)	Raman shift (cm^−1^)	Intensity (a.u.)	Raman shift (cm^−1^)	Intensity (a.u.)	Raman shift (cm^−1^)	Intensity (a.u.)	Raman shift (cm^−1^)	Intensity (a.u.)
D	1356	0.158	1350	0.046	1357	0.041	1312	0.318	1312	0.455	1320	0.641
G	1582	0.954	1570	0.976	1569	0.949	1578	0.898	1576	0.872	1577	0.846
D’	1620	0.040	1610	0.016	1598	0.025	1610	0.095	1608	0.188	1608	0.257
2D	2692	0.265	2677	0.247	2673	0.175	2611	0.093	2617	0.062	2613	0.074
							2647	0.114	2645	0.080	2646	0.070
D + G	2728	0.438	2710	0.261	2709	0.284	2774	0.028	2772	0.064	-	-

**Table 4:** Rheometric parameters of FKM and FKM-based composites.

Sample	Parameters			
	*M* *_L_*	*M* *_H_*	*t*_2_ (min)	*t*_90_ (min)
FKM	0.224 ± 0.005	1.101 ± 0.068	2:38	7:01
FKMG	0.227 ± 0.021	1.376 ± 0.092	2:02	6:06
FKMGN5	0.287 ± 0.003	1.694 ± 0.110	2:10	6:50
FKMGN15	0.253 ± 0.006	1.434 ± 0.085	2:10	6:32

**Table 5:** Tensile mechanical properties, compression set and hardness of FKM and FKM-based composites. Additionally, Property Improvement percentage (PI) was added for each property with the exception of the Compression SET.

Sample					Tensile Test								
	Modulus (MPa)						Tensile Strength (MPa)	PI (%)	Elongation at Break (%)	PI (%)	Hardness (Shore A)	PI (%)	Compression SET (%)
	E50	PI (%)	E100	PI (%)	E300	PI (%)							
FKM	0.69 ± 0.16	-	1.21 ± 0.13	-	4.40 ± 0.38	-	8.81 ± 0.94	-	477 ± 46	-	51.6 ± 1.6	-	2.8 ± 0.4
FKMG	1.47 ± 0.23	113	2.84 ± 0.12	135	7.0 ± 0.11	59	8.90 ± 0.81	1	383 ± 32	−20	55.7 ± 3.2	8	11.6 ± 0.5
FKMGN5	0.82 ± 0.03	18	2.47 ± 0.07	104	8.85 ± 0.48	101	9.68 ± 0.98	10	330 ± 18	−30	57.6 ± 2.0	12	10.7 ± 0.4
FKMGN15	0.77 ± 0.01	12	2.30 ± 0.01	90	7.87 ± 0.06	79	9.42 ± 0.81	7	361 ± 29	−24	64.6 ± 3.1	25	11.0 ± 2.3

**Table 6:** Glass transition temperature, damping factor maximum and Full Width at Half Maximum (FWHM) for FKM-based material.

Sample	T_g_ (°C)	tanδ_max_	FWHM (°C)
FKM	4.2 ± 0.5	1.434 ± 0.0001	16.6 ± 0.5
FKMG	5.1 ± 0.5	1.316 ± 0.0001	17.6 ± 0.5
FKMGN05	4.8 ± 0.5	1.097 ± 0.0001	19.7 ± 0.5
FKMGN15	4.9 ± 0.5	1.197 ± 0.0001	18.3 ± 0.5

**Table 7:** Thermogravimetric and Dynamic mechanical analysis parameters of FKM and FKM composites.

Sample	^a^ T_10_ (°C)	^b^ T_50_ (°C)	^c^ T_max_ (°C)	Residual Mass (%)	^d^ T_g_ (°C)	^e^ T_g_ (°C)
FKM	430.9 ± 0.1	470.3 ± 0.1	474.8 ± 0.1	8.8 ± 0.01	−17.2 ± 0.1	4.2 ± 0.1
FKMG	433.1 ± 0.1	472.9 ± 0.1	476.9 ± 0.1	18.8 ± 0.01	−17.1 ± 0.1	5.1 ± 0.1
FKMGN5	436.9 ± 0.1	475.5 ± 0.1	477.5 ± 0.1	19.3 ± 0.01	−17.5 ± 0.1	4.8 ± 0.1
FKMGN15	435.2 ± 0.1	476.3 ± 0.1	479.5 ± 0.1	16.5 ± 0.01	−17.0 ± 0.1	4.9 ± 0.1

^a^ Temperature of 10% mass loss. ^b^ Temperature of 50% mass loss. ^c^ Maximum degradation temperature. ^d^ Glass transition temperature (T_g_) determined by DSC. ^e^ Glass transition temperature (T_g_) determined by DMA.

**Table 8:** Thermal response values of FKM and FKM-based composites.

Sample	Distance (cm)	Power	Temperature (°C)		
			1 min	5 min	10 min (°C)
FKM	2	1.0 ± 0.1	18.2 ± 0.5	18.3 ± 0.5	18.2 ± 0.5
		5.0 ± 0.1	30.0 ± 0.5	44.4 ± 0.5	46.3 ± 0.5
		10.0 ± 0.1	57.3 ± 0.5	70.1 ± 0.5	71.7 ± 0.5
	5	1.0 ± 0.1	21.2 ± 0.5	21.0 ± 0.5	21.3 ± 0.5
		5.0 ± 0.1	26.7 ± 0.5	30.9 ± 0.5	31.7 ± 0.5
		10.0 ± 0.1	31.2 ± 0.5	37.3 ± 0.5	40.5 ± 0.5
FKMG	2	1.0 ± 0.1	23.4 ± 0.5	23.1 ± 0.5	23.5 ± 0.5
		5.0 ± 0.1	38.0 ± 0.5	45.6 ± 0.5	46.8 ± 0.5
		10.0 ± 0.1	57.3 ± 0.5	69.2 ± 0.5	71.3 ± 0.5
	5	1.0 ± 0.1	23.0 ± 0.5	22.6 ± 0.5	22.8 ± 0.5
		5.0 ± 0.1	23.0 ± 0.5	31.6 ± 0.5	32.4 ± 0.5
		10.0 ± 0.1	33.5 ± 0.5	38.8 ± 0.5	40.7 ± 0.5
FKMGN5	2	1.0 ± 0.1	22.3 ± 0.5	22.7 ± 0.5	22.8 ± 0.5
		5.0 ± 0.1	42.2 ± 0.5	48.7 ± 0.5	51.5 ± 0.5
		10.0 ± 0.1	63.0 ± 0.5	75.0 ± 0.5	76.5 ± 0.5
	5	1.0 ± 0.1	22.7 ± 0.5	22.6 ± 0.5	22.7 ± 0.5
		5.0 ± 0.1	29.5 ± 0.5	33.6 ± 0.5	35.0 ± 0.5
		10.0 ± 0.1	36.1 ± 0.5	41.5 ± 0.5	44.4 ± 0.5
FKMGN15	2	1.0 ± 0.1	22.0 ± 0.5	22.0 ± 0.5	21.8 ± 0.5
		5.0 ± 0.1	31.1 ± 0.5	39.3 ± 0.5	40.6 ± 0.5
		10.0 ± 0.1	44.5 ± 0.5	57.8 ± 0.5	59.7 ± 0.5
	5	1.0 ± 0.1	21.3 ± 0.5	21.3 ± 0.5	21.8 ± 0.5
		5.0 ± 0.1	31.4 ± 0.5	32.8 ± 0.5	33.5 ± 0.5
		10.0 ± 0.1	32.7 ± 0.5	39.8 ± 0.5	42.0 ± 0.5

## Data Availability

The data presented in this study are available on request from the corresponding author due to privacy concerns.
